# Impact of classical and basal-like molecular subtypes on overall survival in resected pancreatic cancer in the SPACIOUS-2 multicentre study

**DOI:** 10.1093/bjs/znac272

**Published:** 2022-08-18

**Authors:** J Annelie Suurmeijer, Eline C Soer, Mark P G Dings, Yongsoo Kim, Marin Strijker, Bert A Bonsing, Lodewijk A A Brosens, Olivier R Busch, Jesse V Groen, Johannes B Halfwerk, Robbert A E Slooff, Hanneke W M van Laarhoven, I Quintus Molenaar, G Johan A Offerhaus, Hans Morreau, Marc J van de Vijver, Arantza Fariña Sarasqueta, Joanne Verheij, Marc G Besselink, Maarten F Bijlsma, Frederike Dijk, for the Dutch Pancreatic Cancer Group, Livia de Guerre

**Affiliations:** Department of Surgery, Amsterdam UMC, location University of Amsterdam, Amsterdam, The Netherlands; Cancer Center Amsterdam, Amsterdam, The Netherlands; Cancer Center Amsterdam, Amsterdam, The Netherlands; Department of Pathology, Amsterdam UMC, location University of Amsterdam, Amsterdam, The Netherlands; Cancer Center Amsterdam, Amsterdam, The Netherlands; Center for Experimental and Molecular Medicine, Laboratory for Experimental Oncology and Radiobiology, Amsterdam UMC, location University of Amsterdam, Amsterdam, The Netherlands; Cancer Center Amsterdam, Amsterdam, The Netherlands; Department of Pathology, Amsterdam UMC, location Vrije Universiteit Amsterdam, Amsterdam, The Netherlands; Department of Surgery, Amsterdam UMC, location University of Amsterdam, Amsterdam, The Netherlands; Cancer Center Amsterdam, Amsterdam, The Netherlands; Department of Surgery, Leiden University Medical Center, Leiden, The Netherlands; Department of Pathology, University Medical Center Utrecht, Utrecht, The Netherlands; Faculty of Medicine, Utrecht University, Utrecht, The Netherlands; Department of Surgery, Amsterdam UMC, location University of Amsterdam, Amsterdam, The Netherlands; Cancer Center Amsterdam, Amsterdam, The Netherlands; Department of Surgery, Leiden University Medical Center, Leiden, The Netherlands; Cancer Center Amsterdam, Amsterdam, The Netherlands; Department of Pathology, Amsterdam UMC, location University of Amsterdam, Amsterdam, The Netherlands; Department of Surgery, University Medical Center Utrecht, Utrecht, The Netherlands; Cancer Center Amsterdam, Amsterdam, The Netherlands; Department of Medical Oncology, Amsterdam UMC, location University of Amsterdam, Amsterdam, The Netherlands; Regional Academic Cancer Center Utrecht, Department of Hepato-Pancreato-Biliary Surgery, St. Antonius Hospital Nieuwegein & University Medical Center Utrecht, Utrecht, The Netherlands; Department of Pathology, University Medical Center Utrecht, Utrecht, The Netherlands; Faculty of Medicine, Utrecht University, Utrecht, The Netherlands; Department of Pathology, Leiden University Medical Center, Leiden, The Netherlands; Cancer Center Amsterdam, Amsterdam, The Netherlands; Department of Pathology, Amsterdam UMC, location University of Amsterdam, Amsterdam, The Netherlands; Cancer Center Amsterdam, Amsterdam, The Netherlands; Department of Pathology, Amsterdam UMC, location University of Amsterdam, Amsterdam, The Netherlands; Cancer Center Amsterdam, Amsterdam, The Netherlands; Department of Pathology, Amsterdam UMC, location University of Amsterdam, Amsterdam, The Netherlands; Department of Surgery, Amsterdam UMC, location University of Amsterdam, Amsterdam, The Netherlands; Cancer Center Amsterdam, Amsterdam, The Netherlands; Cancer Center Amsterdam, Amsterdam, The Netherlands; Center for Experimental and Molecular Medicine, Laboratory for Experimental Oncology and Radiobiology, Amsterdam UMC, location University of Amsterdam, Amsterdam, The Netherlands; Cancer Center Amsterdam, Amsterdam, The Netherlands; Department of Pathology, Amsterdam UMC, location University of Amsterdam, Amsterdam, The Netherlands

## Abstract

**Background:**

The recently identified classical and basal-like molecular subtypes of pancreatic cancer impact on overall survival (OS). However, the added value of routine subtyping in both clinical practice and randomized trials is still unclear, as most studies do not consider clinicopathological parameters. This study examined the clinical prognostic value of molecular subtyping in patients with resected pancreatic cancer.

**Methods:**

Subtypes were determined on fresh-frozen resected pancreatic cancer samples from three Dutch centres using the Purity Independent Subtyping of Tumours classification. Patient, treatment, and histopathological variables were compared between subtypes. The prognostic value of subtyping in (simulated) pre- and postoperative settings was assessed using Kaplan–Meier and Cox regression analyses.

**Results:**

Of 199 patients with resected pancreatic cancer, 164 (82.4 per cent) were classified as the classical and 35 (17.6 per cent) as the basal-like subtype. Patients with a basal-like subtype had worse OS (11 *versus* 16 months (HR 1.49, 95 per cent c.i. 1.03 to 2.15; *P* = 0.035)) than patients with a classical subtype. In multivariable Cox regression analysis, including only clinical variables, the basal-like subtype was a statistically significant predictor for poor OS (HR 1.61, 95 per cent c.i. 1.11 to 2.34; *P* = 0.013). When histopathological variables were added to this model, the prognostic value of subtyping decreased (HR 1.49, 95 per cent c.i. 1.01 to 2.19; *P* = 0.045).

**Conclusion:**

The basal-like subtype was associated with worse OS in patients with resected pancreatic cancer. Adding molecular classification to inform on tumor biology may be used in patient stratification.

## Teaser

The classical and basal-like molecular subtypes of pancreatic adenocarcinoma have prognostic value, but the benefit of these subtypes in daily clinical practice remains unclear. This multicentric study of 199 patients after resection for pancreatic cancer found that molecular subtype is prognostic in the preoperative setting but less so in the postoperative setting when histopathological parameters are available.

## Introduction

Pancreatic cancer is predicted to become the second most common cause of cancer death by 2030; however, the treatment options remain limited and survival remains poor^1,2^. Prognosis relies mainly on clinical staging and histopathological assessment^3^. Molecular subtyping of pancreatic cancer could help stratify patients both in clinical practice and in randomized trials, ultimately leading to optimized treatment algorithms^4,5^. In recent years, several groups have identified two subtypes of pancreatic cancer that show high concordance in their gene expression profiles: a classical subtype and a more aggressive, basal-like subtype^6–11^. The basal-like subtype is associated with worse survival and is characterized by the high expression of genes related to epithelial to mesenchymal transition, a process by which tumour cells gain migratory and invasive properties^6^. Classical and basal-like subtypes have also been established in other cancers (e.g. head and neck, urothelial, and breast)^12,13^.

Until recently, the implementation of molecular subtyping in clinical practice was seen as costly and cumbersome, requiring fresh-frozen tumour tissue with sufficient tumour cellularity and laborious RNA sequencing. The recently published Purity Independent Subtyping of Tumours (PurIST) classifier requires expression analysis of only 16 genes to predict the previously established Moffitt basal-like and classical subtypes for each sample individually^14^. This classifier showed a strong correspondence between subtypes from previously published discovery cohorts (i.e. Bailey, Moffitt, and Collinson), with an overall consensus area under the curve value of 0.993^9,10,15–19^. Furthermore, the application of PurIST demonstrated the significant agreement of subtypes between matched pairs of bulk fresh-frozen samples, formalin-fixed tissue, and fine-needle aspiration^14^. These advances are likely to reduce the overall costs and enable the implementation of subtyping in daily clinical practice. However, the added prognostic value of molecular subtyping compared to readily available clinicopathological variables is currently unclear, as most previous studies did not consider these variables^9–11,14,19,20^.

The aim of this study was to examine the clinical prognostic benefit of the molecular subtypes of pancreatic cancer in a real-world multicentre cohort of patients resected for pancreatic cancer.

## Methods

### Patient selection

Fresh-frozen tumour tissue derived from resected pancreatic ductal adenocarcinoma (henceforth ‘pancreatic cancer’) between 1993 and 2015 was collected from the cryoarchives of the Departments of Pathology at the Amsterdam UMC, University Medical Center Utrecht, and Leiden University Medical Center (the SPACIOUS consortium). The current study builds on the previous SPACIOUS-1 cohort study^7^. This study was approved by the Amsterdam UMC Institutional Review Board (METC_A1 15.0122). Resection specimens were retrospectively collected in accordance with ethical guidelines (‘Code for Proper Secondary Use of Human Tissue in the Netherlands’ (Dutch Federation of Medical Scientific Societies)). For prospectively collected material, informed consent was obtained from all patients in accordance with our hospital’s ethical guidelines (METC 2018_181). Samples from patients with metastatic disease were excluded. Clinicopathological data were obtained from the departments of surgery and pathology.

### Sample information and molecular classification

All specimens were snap-frozen in liquid nitrogen and stored at −80°C. Five dedicated pancreatic pathologists (A.F.S., J.V., L.A.A.B., H.M., and G.J.A.O.) retrospectively assessed the specimens used for RNA extraction for tumour cell percentage and diagnosis. The PurIST classifier was used on this cohort, categorizing tumours as either the classical or basal-like subtype based on RNA sequencing data (*Fig. S1*)^14^. To obtain high-quality data, only samples with invasive pancreatic cancer and a tumour cell percentage of more than 30 per cent were included for analysis^19^. Samples with poor-quality RNA were excluded. More information on the processing, revision of the samples, and subtype label assignment can be found in the *supplementary material*.

### Definitions

Patients were stratified according to molecular subtype. Clinical parameters included age, sex, and ASA grade. The pathological parameters were those in the minimum data set for histological reporting (i.e. grade of differentiation, tumour size, lymphovascular and perineural invasion, lymph node status, and resection margin status)^21^. Pathological reports were retrospectively reclassified according to the eighth edition of the AJCC staging criteria^3^.

### Outcome measure

The primary outcome was overall survival (OS), defined as the time between surgery and date of death or date of last follow-up, according to the National Personal Records Database.

### Statistical analysis

Continuous data were expressed as medians with interquartile ranges (i.q.r.) and tested using the Mann–Whitney *U* test. Categorical data were presented as frequencies with percentages and analysed using the χ^2^ test or Fisher’s exact test for categories containing small numbers. The null hypothesis was rejected if the *P* value was ≤ 0.050. Margin status was defined by margin: R0 margin 1 mm or more; and R1 less than 1 mm. The anterior surface was not considered for R status as this remains a point of discussion^22^. Categories with numbers less than five were dichotomized based on a clinically relevant cut-off value.

The date of the last follow-up was October 2021. Patients who died as a result of postoperative complications were excluded. Kaplan–Meier survival analysis was used to assess differences in survival between the classical and basal-like subtypes. To demonstrate whether subtype was associated with OS, multivariable Cox proportional hazards regression analysis with backward selection was performed in two different manners: considering only preoperative clinical variables (i.e. sex, age, and ASA grade), to simulate a preoperative setting; and a postoperative setting considering all clinical and pathological variables. Backward selection was performed by removing the highest *P* value in the model until all remaining variables had a *P* value < 0.1. Potential prognostic factors were age (continuous), sex (male, female), neoadjuvant therapy (no, yes), tumour location (head, corpus/tail), tumour diameter (continuous), margin status (R0, R1), differentiation grade (well/moderate, poor), perineural growth (no, yes), vasoinvasive growth (no, yes), lymph node ratio (continuous), and adjuvant therapy (minimum of two cycles; no, yes). To appraise the potential effects of preoperative treatment on molecular subtype and pathology-based variables, a sensitivity analysis was performed excluding patients with preoperative treatment. Statistical analyses were performed using R-studio version 3.6.1.

## Results

Overall, 560 tumour samples of resected pancreatic cancer were obtained from 560 patients. Excluded were 324 (57.9 per cent) samples with a tumour cell percentage of less than 30 per cent, 21 (3.8 per cent) samples after revision for diagnosis, and 16 samples (2.9 per cent) based on poor quality of RNA, leaving 199 (35.5 per cent) samples from 199 patients available for the final analysis. Kaplan–Meier analysis of all included samples stratified for inclusion or exclusion did not show a difference in survival.

### Clinicopathological characteristics

The baseline, treatment, and histopathological characteristics of patients with the classical and basal-like tumour subtypes are shown in *Table 1*. Of the 199 included tumour samples, 164 (82.4 per cent) were classified as classical and 35 (17.6 per cent) as basal-like pancreatic cancer. Poor differentiation grade was associated with the basal-like subtype (odds ratio (OR) 2.27, 95 per cent c.i. 1.04 to 5.28; *P* = 0.042). There were neither differences in baseline characteristics nor in the administration of adjuvant therapy between the two groups.

**Table 1:** Baseline and treatment characteristics of the classical and basal-like molecular subtype in resected pancreatic cancer

	Overall	Classical subtype	Basal-like subtype	*P* value*
**Total**	199 (100)	164 (84.4)	35 (17.6)	
**Age at diagnosis†**	67.0 (59.0–73.0)	66.0 (59.0–71.2)	69.0 (59.0–74.5)	0.279
**Sex (female)**	95 (47.7)	83 (50.6)	12 (34.3)	0.079
**ASA score**				0.839
	1	44 (22.3)	37 (22.8)	7 (20.0)
2	115 (58.4)	93 (57.4)	22 (62.9)	
≥3	38 (19.3)	32 (19.8)	6 (17.1)	
Missing	2	2	0	
**Tumour location (head)**	171 (85.9)	141 (86.0)	30 (85.7)	>0.999
**Preoperative therapy**	3 (1.5)	3 (1.8)	0 (0.0)	>0.999
**Year of surgery‡**	2012 (1993–2015)	2011 (1993–2015)	2013 (1993–2015)	0.089
**Type of surgery**				0.699
Pancreatoduodenectomy	174 (87.9)	144 (88.3)	30 (85.7)	
Distal pancreatectomy	22 (11.1)	17 (10.4)	5 (14.3)	
Total pancreatectomy	2 (1.0)	2 (1.2)	0 (0.0)	
Missing	1	1	0	
**Margin status (R0)**	102 (51.3)	82 (50.0)	20 (57.1)	0.443
**Differentiation grade (poor)**	106 (53.3)	81 (49.4)	25 (71.4)	0.018
**Perineural growth**	156 (80.4)	125 (78.1)	31 (91.2)	0.082
Missing	5	4	1	
**Lymphovascular growth**	92 (46.9)	72 (44.7)	20 (57.1)	0.182
Missing	3	3	0	
**Lymph node ratio†**	0.2 (0.1–0.4)	0.2 (0.1–0.3)	0.2 (0.0–0.4)	0.803
Missing	2	2	0	
**T stage (AJCC 8th)**				0.702
1	31 (15.7)	27 (16.6)	4 (11.4)	
2	128 (64.6)	105 (64.4)	23 (65.7)	
3	39 (19.7)	31 (19.0)	8 (22.9)	
Missing	1	1	0	
**N stage (AJCC 8th)**				0.687
0	41 (20.6)	32 (19.5)	9 (25.7)	
1	95 (47.7)	80 (48.8)	15 (42.9)	
2	63 (31.7)	52 (31.7)	11 (31.4)	
**Adjuvant therapy**				0.567
	151 (76.3)	123 (75.5)	28 (80.0)	
Missing	1	1	0	
**Type of adjuvant therapy**				0.515
None	45 (23.0)	39 (24.1)	6 (17.6)	
Gemcitabine	147 (75.0)	120 (74.1)	27 (79.4)	
Capecitabine	4 (2.0)	3 (1.9)	1 (2.9)	
Missing	3	2	1	
**Status (diseased)**	191 (96.0)	156 (95.1)	35 (100.0)	0.355

Data are provided as *n* (%) unless otherwise stated. *Mann–Whitney *U* test; Pearson's χ^2^ test; Fisher's exact test. †Median (interquartile range). ‡Median (total range).

### Survival analysis

**Fig. 1:**
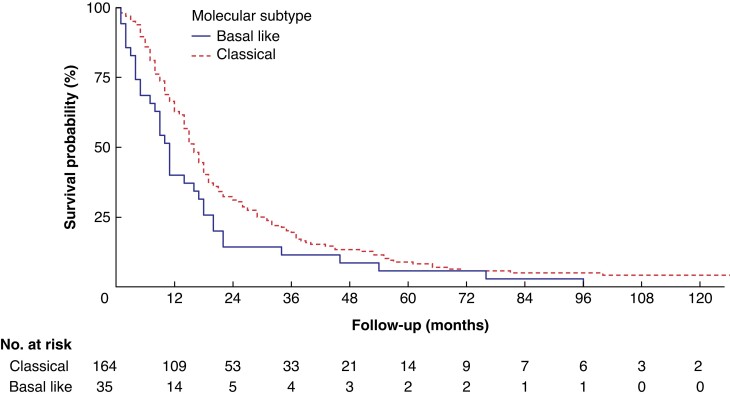
Overall survival after resection for pancreatic cancer, stratified by molecular subtype

At the time of the last follow-up, eight (4 per cent) patients with the classical subtype were alive and no (0 per cent) patients with the basal-like subtype were alive. The median (i.q.r.) follow-up of patients alive at the last follow-up was 106 (i.q.r. 48–158) months. Patients with a basal-like tumour had a worse median OS of 11 *versus* 16 months for those with the classical subtype (HR 1.49, 95 per cent c.i. 1.03 to 2.15; *P* = 0.035) (*Fig. 1*).

Variables associated with survival in univariable Cox regression analysis were age, tumour diameter, margin status, differentiation grade, perineural growth, lymphovascular invasion, lymph node ratio, and molecular subtype (*Tables 2 and 3*). In multivariable Cox regression analysis including only the clinical parameters available at the time of surgery, the basal-like subtype was a predictor of poor survival (HR 1.61, 95 per cent c.i. 1.11 to 2.34; *P* = 0.013). In multivariable Cox regression analysis including all relevant clinical and histopathological parameters, the basal-like subtype remained a significant predictor of poor OS (HR 1.49, 95 per cent c.i. 1.01 to 2.19; *P* = 0.045). The most important pathological tumour characteristics predicting OS were lymph node ratio, poor tumour differentiation, and margin status (R1).

**Table 2:** Clinical predictors of survival in uni- and multivariable regression analysis

	Univariable analysis			Multivariable analysis		
	HR*	95% c.i.	*P* value	HR	95% c.i.	*P* value†
**Age**	1.02	1.00–1.03	0.049	1.02	1.00–1.03	0.036
**Sex (female)**	1.23	0.61–1.09	0.160			
**Tumour location (pancreatic tail)**	0.75	0.50–1.14	0.179	0.69	0.45–1.06	0.088
**ASA grade**						
1	Reference					
2	1.41	0.97–2.04	0.069			
≥3	1.44	0.91–2.27	0.117			
**Molecular subtype (basal-like)**	1.49	1.03–2.15	0.035	1.61	1.11–2.34	0.013

*Hazard ratios (HR) in Cox regression analyses on survival. †Analysed in 199 complete cases via multivariable Cox regression analysis with backward selection until predictors with a *P* value <0.10 remained.

**Table 3:** Clinicopathological predictors of overall survival in uni- and multivariable analysis

	Univariable analysis			Multivariable analysis		
	HR*	95% c.i.	*P* value	HR*	95% c.i.	*P* value†
**Age**	1.02	1.00–1.03	0.049	1.03	1.01–1.05	<0.001
**Sex (female)**	1.23	0.61–1.09	0.160			
**Tumour location (tail)**	0.75	0.50–1.14	0.179			
**Neoadjuvant therapy**	0.73	0.23–2.30	0.597			
**Tumour diameter**	1.11	1.01–1.22	0.029			
**Margin status (R1)**	1.60	1.20–2.14	0.001	1.52	1.12–2.08	0.007
**Differentiation grade (poor)**	1.61	1.21–2.15	0.002	1.70	1.25–2.31	<0.001
**Perineural growth**	1.49	1.03–2.15	0.034	1.61	1.07–2.42	0.022
**Lymphovascular invasion**	1.59	1.19–2.12	0.002	1.35	0.97–1.88	0.078
**Lymph node ratio**	2.99	1.67–5.35	<0.001	3.30	3.30–6.45	<0.001
**Adjuvant therapy**	0.75	0.54–1.05	0.097	0.66	0.45–0.96	0.031
**Molecular subtype (basal-like)**	1.49	1.03–2.15	0.035	1.49	1.01–2.19	0.045

*Hazard ratios in Cox regression analyses on survival. †Analysed in 191 complete cases via multivariable Cox regression analysis with backward selection until predictors with a *P* value <0.10 remained.

Subgroup analysis in 106 patients with a poor differentiation grade showed no statistical difference in OS in the basal-like subtype (8 *versus* 14 months; *P* = 0.440 (*Fig. S2*)) compared with the classical subtype. Analysis of the prognostic value of TN staging and margin status in both subtypes showed that, unlike in the classical subtype, T stage (HR 1.52, 95 per cent c.i. 0.34 to 1.30; *P* = 0.229), N stage (HR 1.48, 95 per cent c.i. 0.95 to 2.30; *P* = 0.084), and margin status (HR 1.66, 95 per cent c.i. 0.80 to 3.42; *P* = 0.171) had no prognostic value in the basal-like subtype (*Table S1*).

After sensitivity analysis, which excluded patients after preoperative systemic therapy, the association between basal-like subtype and poor OS in multivariable regression analysis remained (HR 1.48, 95 per cent c.i. 1.00 to 2.18; *P* = 0.048 (*Table S2*)).

## Discussion

This multicentric study confirmed the association of the basal-like subtype with poor OS in patients with resected pancreatic cancer. The prognostic value was less when other histopathological variables were considered. Based on this, molecular classification will likely add information on the biology of a tumour and therefore could have a role in future patient stratification.

Over the last decade, many groups have used unsupervised class discovery for the molecular classification of pancreatic cancer^7,11,19,20,23–25^. Although the number of identified subtypes varied, a basal-like subtype was consistently found (*Fig. S1*). Although consensus on a universal classifier is lacking, most groups now agree on a two-tier classification of pancreatic cancer. The poor prognosis of the basal-like subtype is well established, but little is known about the added value when considering traditional clinicopathological variables. A few discovery cohorts used multivariable Cox analysis to assess whether subtype was an independent predictor for OS, but information on the methods used to obtain and completeness of clinical and histopathological data was mostly lacking^7,10,23–25^.

A recent international study containing tumour samples from 442 patients with pancreatic cancer concluded that molecular subtype was a strong prognostic factor and should be considered when staging patients with resected pancreatic cancer^4^. This study used a subtyping method that is more difficult to perform on small biopsy material, hindering its use in the preoperative or metastatic setting. For patients with the basal-like subtype (25.3 per cent), prognosis was extremely poor, irrespective of the T- and N-stage and margin status, with an OS of 14.9 months^4^. This finding was confirmed in the current multicentric study, indicating that the power of the histopathological variables is limited in the basal-like subgroup as it is in itself a predictor of aggressive tumour behaviour.

In the present multicentric cohort, 35 patients (nearly 18 per cent) had a basal-like subtype, a smaller proportion than found in other studies^7,9,10^. This suggests that the PurIST classifier predicts the basal subtype with higher specificity. However, the high degree of intratumour heterogeneity known to exist in pancreatic cancer is not considered in these binary classification systems. Although continuous classifications are proposed and a probability score is provided in PurIST, a cut-off value remains essential for any treatment choice in clinical practice^26^.

Of all the prognostic clinicopathological variables, only poor differentiation grade was associated with the basal-like subtype. Poor differentiation is considered a measure of aggressive tumour behaviour, and its association is to be expected. Surprisingly, the other established histopathological variables that predict aggressive tumour behaviour (i.e. tumour size, lymph node ratio, lymphovascular, and perineural invasion) did not show a significant association with the basal subtype. It remains unclear whether this is because of the sample size or whether it implies that subtyping confers prognostic information that is supplementary to the standard histopathological assessment. During the range of time in which this cohort was collected, pathology reporting and sampling has considerably improved, resulting in a more accurate detection of lymphovascular and perineural invasion. This should be studied in future large-scale studies.

The results of this study should be interpreted in the light of certain limitations. Firstly, this cohort had a long inclusion period. However, treatment strategies were very similar during the inclusion time and we therefore believe the heterogeneity of this cohort is relatively limited. Secondly, most patients were included before the era of preoperative therapy, which is not a reflection of current practice, especially in borderline resectable disease. Similarly, none of the patients received preoperative or adjuvant FOLFIRINOX chemotherapy. However, it is unclear whether and to what extent preoperative therapy affects the clinical impact of subtyping^15^. This should be further investigated. Thirdly, only data from high-cellularity samples were available, and only 39 per cent of the tumours were included. Because pancreatic cancer is, in general, characterized by low tumour cellularity, this is a common problem in studies of the molecular subtyping^19^. As the PurIST classifier only uses rank-transformed, relative expression of tumour cell-intrinsic gene pairs, subtype can be determined in samples with a lower cancer cell percentage. Of 13 samples initially excluded from our cohort due to low cellularity, RNA sequencing data were available. In fact, the PurIST classification could be applied to these samples, discovering 12 classical subtypes and one basal-like subtype. However, validation is needed in a larger cohort of low cellularity samples, such as fresh-frozen paraffin-embedded biopsies. Finally, although our sample size was relatively large for this study type, the number of basal-like samples remained small. Therefore, we could not exclude a type II error regarding the primary endpoint.

The prognostic and predictive value of the basal-like subtype remains to be validated in patients receiving preoperative therapy and in the metastatic setting. Future studies may benefit from the recent advancements in RNA sequencing and apply subtyping on lower quantities and quality of tumour tissue^11,23^. Currently, there are only few predictive factors to guide the choice of preoperative and adjuvant systemic therapy in patients with pancreatic cancer. The basal-like subtype, based on tumour-intrinsic biological features, has been proven to be robust and shows promise in this area^27,28^. However, an important step before implementing subtyping in randomized trials on systemic treatment is to achieve consensus on which classifier to use. Such consensus will facilitate better comparison across trials, and enable researchers to study the predictive value of gene signatures and patient management and outcomes in more detail^29^. Ultimately, this should lead to improved patient stratification and, as a result, treatment outcomes for patients with pancreatic cancer.

## Collaborators

Livia de Guerre (University Medical Center Utrecht, Utrecht, the Netherlands).

## Funding

This work was supported by a KWF Dutch Cancer Society grant to M.J.V., O.R.B., and H.W.L. (UVA 2014–6803), and by a Deltaplan Alvleesklierkanker grant to J.A.S. and M.G.B. The funders were not involved in the study design or drafting of the manuscript.

## Supplementary Material

znac272_Supplementary_Data

## Data Availability

The authors had complete access to the study data that support this publication. All relevant data are within the paper and its *supplementary material* files. Data, analytic methods, and study materials can be made available to other researchers on reasonable request.
