# Supplementary material for: Impact of classical and basal-like molecular subtypes on overall survival in resected pancreatic cancer in the SPACIOUS-2 multicentre study
Source: Br J Surg. 2022 Aug 18;109(11):1150–5. doi: 10.1093/bjs/znac272 (PMC10364758; doi:10.1093/bjs/znac272)
Supplement: znac272_Supplementary_Data [file znac272_supplementary_data.zip › Supplementary_material.docx]

**Supplementary METHODS.**

Information on revision, selection, and processing of samples

Frozen sections (5 µm) were stained with hematoxylin and eosin (HE) and all slides were examined by pancreatic pathologists (J.V. and A.F.S. for Amsterdam UMC samples; L.A.B and G.J.O for UMC Utrecht samples; A.F.S. and J.M. for Leiden UMC samples) to assess presence of invasive tumour and tumour cell percentage of >30 per cent. The pathology report and all formalin-fixed paraffin-embedded (FFPE) tumour slides were revised to ascertain the primary origin of the tumour as pancreatic cancer.

Preparation of libraries and processing for RNA-seq

For RNA isolation, 30 sections of 20 μm were cut, and RNA was isolated using RNABee (Bio-Connect, Huissen, the Netherlands) and the RNeasy Mini kit (Qiagen, Hilden, Germany) according to the manufacturer’s instructions. In most samples, the RNA Integrity Number (RIN) was 7 or higher, as evaluated by BioAnalyzer (Agilent, Santa Clara, CA, USA), median RIN = 8.2. Of nine samples, the RIN value was less than 7, but this was not apparent from principal component analysis (PCA) of the gene expression profiles. The samples were DNAse-treated. RNA was amplified using the Total Prep RNA Amplification Kit (Illumina, San Diego, CA, USA). Poly A-enriched libraries were synthesized using the TruSeq RNA Library Prep kit and sequenced in three batches (Illumina HiSeq2500). All sequencing data were quality-controlled using FastQC42 and were found to be of high quality. RNA-Seq reads were aligned to the human reference genome (GRCh38) using Tophat2 (V2.1.043) with default parameters, retaining only uniquely mapped reads. Gene expression levels were estimated using Cufflinks (V2.2.1), with default parameters and Gencode V19 for gene annotation, masking rRNAs, tRNAs, and chromosome M. The resulting gene expression profiles, measured by RPKM (reads per kilobase of transcript per million mapped reads) were log2-transformed. Non-biological batch effects were inspected using PCA, and corrections were made using Combat44. Subsequent analyses were performed on the batch-corrected dataset.

Assignment of subtype labels

Classification labels were assigned using the PurIST classifier10. In brief, the R package Switchbox was used to obtain the TSP votes per sample. Votes in favor of the Basal-like subtype were matrix factorized by their penalized regression coefficients and then subtracted by 6.81. Samples with a PurIST score higher than 0 were considered Basal-like; otherwise, they were considered Classical.

**TABLE S1.** Subgroup analysis of T and N stage and resection margin status (R) on survival after resection of pancreatic cancer

|  | Univariable analysis | | |
| --- | --- | --- | --- |
|  | **HR^*^** | **95% CI^Ϯ^** | **P Value** |
| Basal-like (N=35) |  |  |  |
| T stage | 1.516 | 0.34-1.30 | 0.229 |
| N stage | 1.476 | 0.95-2.30 | 0.084 |
| R stage | 1.659 | 0.80-3.42 | 0.171 |
| Classical (N=164) |  |  |  |
| T stage | 1.480 | 1.13-1.94 | **0.005** |
| N stage | 1.548 | 1.21-1.98 | **<0.001** |
| R stage | 1.591 | 1.15-2.20 | **0.005** |
| * Hazard Ratios in Cox regression analyses on survival  Ϯ 95% Confidence interval | | | |

**Table S2.** Clinicopathological predictors of survival in patients with upfront-resected pancreatic cancer in multivariable cox regression analysis

|  | HR^*^ | 95% CI^Ϯ^ | P-value^§^ |
| --- | --- | --- | --- |
| Age | 1.03 | 1.01-1.05 | **0.001** |
| Margin status (R1) | 1.53 | 1.12-2.09 | **0.008** |
| Differentiation grade (poor) | 1.72 | 1.26-2.34 | **0.001** |
| Perineural growth | 1.59 | 1.06-2.40 | **0.025** |
| Vasoinvasive growth | 1.36 | 0.97-1.90 | 0.074 |
| Lymph node ratio | 3.31 | 1.69-6.48 | **<0.001** |
| Adjuvant therapy | 0.66 | 0.46-0.97 | **0.032** |
| Molecular subtype (basal-like) | 1.48 | 1.00-2.18 | **0.048** |
| * Hazard Ratios in Cox regression analyses on survival  Ϯ 95% Confidence interval  § Analyzed in in 187 complete cases via multivariable cox regression analysis with backward selection until predictors with a p-value <0.1 remained | | | |
